# A Strong Decline in the Incidence of Childhood Otitis Media During the COVID-19 Pandemic in the Netherlands

**DOI:** 10.3389/fcimb.2021.768377

**Published:** 2021-11-01

**Authors:** Saskia Hullegie, Anne G. M. Schilder, Paola Marchisio, Joline L. H. de Sévaux, Alike W. van der Velden, Alma C. van de Pol, Josi A. Boeijen, Tamara N. Platteel, Sara Torretta, Roger A. M. J. Damoiseaux, Roderick P. Venekamp

**Affiliations:** ^1^ Julius Center for Health Sciences and Primary Care, University Medical Center Utrecht, Utrecht University, Utrecht, Netherlands; ^2^ evidENT, Ear Institute, University College London, London, United Kingdom; ^3^ National Institute for Health Research, Hospitals Biomedical Research Centre, University College London, London, United Kingdom; ^4^ Fondazione Istituto di Ricovero e Cura a Carattere Scientifico (IRCCS) Ca’ Granda Ospedale Maggiore Policlinico Pediatric Highly Intensive Care Unit, Milan, Italy; ^5^ Department of Pathophysiology and Transplantation, Università degli Studi di Milano, Milan, Italy; ^6^ Fondazione Istituto di Ricovero e Cura a Carattere Scientifico (IRCCS) Ca’ Granda Ospedale Maggiore Policlinico, Ear Nose Throat (ENT) and Head and Neck Surgery Unit, Milan, Italy; ^7^ Department of Clinical Sciences and Community Health, Università degli Studi di Milano, Milan, Italy

**Keywords:** otitis media, incidence, antibiotic, COVID-19 pandemic, children

## Abstract

**Introduction:**

Recent reports have highlighted the impact of the COVID-19 pandemic on the incidence of infectious disease illnesses and antibiotic use. This study investigates the effect of the pandemic on childhood incidence of otitis media (OM) and associated antibiotic prescribing in a large primary care-based cohort in the Netherlands.

**Material and Methods:**

Retrospective observational cohort study using routine health care data from the Julius General Practitioners’ Network (JGPN). All children aged 0-12 registered in 62 practices before the COVID-19 pandemic (1 March 2019 - 29 February 2020) and/or during the pandemic (1 March 2020 - 28 February 2021) were included. Data on acute otitis media (AOM), otitis media with effusion (OME), ear discharge episodes and associated antibiotic prescriptions were extracted. Incidence rates per 1,000 child years (IR), incidence rate ratios (IRR) and incidence rate differences (IRD) were compared between the two study periods.

**Results:**

OM episodes declined considerably during the COVID-19 pandemic: IR pre-COVID-19 vs COVID-19 for AOM 73.7 vs 27.1 [IRR 0.37]; for OME 9.6 vs 4.1 [IRR 0.43]; and for ear discharge 12.6 vs 5.8 [IRR 0.46]. The absolute number of AOM episodes in which oral antibiotics were prescribed declined accordingly (IRD pre-COVID-19 vs COVID-19: -22.4 per 1,000 child years), but the proportion of AOM episodes with antibiotic prescription was similar in both periods (47% vs 46%, respectively).

**Discussion:**

GP consultation for AOM, OME and ear discharge declined by 63%, 57% and 54% respectively in the Netherlands during the COVID-19 pandemic. Similar antibiotic prescription rates before and during the pandemic indicate that the case-mix presenting to primary care did not considerably change. Our data therefore suggest a true decline as a consequence of infection control measures introduced during the pandemic.

## Introduction

On 11 March 2020, the WHO (World Health Organization, 2020) declared a global pandemic of COVID-19 which enforced many countries to introduce generic infection control measures such as wearing face masks, hand washing, social distancing, working from home and closure of schools/daycare centers. Other than reducing SARS-CoV-2 transmission, these generic measures have likely affected transmission of other respiratory viruses (Fricke et al., 2021; Tang et al., 2021). Since otitis media (OM) is generally preceded by a viral upper respiratory tract infection (URTI) (Schilder et al., 2016), changes in transmission dynamics of these viruses may have had an impact on the incidence of OM. OM, one of the commonest conditions during early childhood and a prime reason for antibiotic prescriptions (Klein, 2000; Monasta et al., 2012; van den Broek d’Obrenan et al., 2014; Tong et al., 2018), consists of a spectrum of diseases, including acute otitis media (AOM), otitis media with effusion (OME), and chronic suppurative otitis media (CSOM). AOM is characterized by the presence of middle ear effusion (MEE) with rapid onset of signs and symptoms of an acute infection such as fever and ear pain (Lieberthal et al., 2013). Approximately 15%-20% of children with AOM present with ear discharge due to a spontaneous tear or perforation of the eardrum (Rovers et al., 2006; Smith et al., 2010). OME is defined by the presence MEE, without signs and symptoms of an acute infection (Rosenfeld et al., 2016). Hearing loss is the most common symptom of OME. CSOM is characterized by chronic inflammation of the middle ear and mastoid mucosa together with a non-intact tympanic membrane and persisting ear discharge (Verhoeff et al., 2006). Several reports from the early phase of the COVID-19 pandemic have suggested a decline in doctor consultations for OM in children (Hatoun et al., 2020; Aldè et al., 2021; Angoulvant et al., 2021; Iannella et al., 2021; Kaur et al., 2021; Torretta et al., 2021; van de Pol et al., 2021). The question is whether the infection control measures or the sudden COVID-19 related changes in health care access and delivery are responsible for these changes. We will address this question by investigating the effect of the COVID-19 pandemic on OM consultations and associated antibiotic prescribing in children in a large primary care cohort in the Netherlands where the general practitioner (GP) is the first point of call (Grobbee et al., 2005) for the management of OM for all children and practices could be contacted for medical advice throughout the pandemic.

## Material and Methods

### Design and Study Population

In this retrospective observational cohort study, data were obtained from the Julius General Practitioners’ Network (JGPN). Its database contains anonymously extracted routine health care data from electronic records from 62 general practices in the Utrecht area (Smeets et al., 2018). All children aged 0-12 registered 1 March 2019 - 29 February 2020 (pre-COVID-19 pandemic) and/or 1 March 2020 - 28 February 2021 (COVID-19 pandemic) were included. The Medical Research Ethics Committee Utrecht has reviewed the study protocol and declared that official ethical approval is not required since this research is outside the scope of the Dutch Medical Research Involving Human Subjects Act (protocol no 21-562/C).

### Data Extraction

From the electronic health records, GP consultations - both face-to-face as well as telephone consultations - of OM (International Classification of Primary Care [ICPC] code H04 (ear discharge); H71 (acute otitis media) H72 (otitis media with effusion; all episodes, irrespective of preceding GP consultation of AOM) and H01 (ear pain) were extracted. A new OM episode started if there was no OM-related GP consultation for 28 days. For each episode, the start date, the child’s age at the start of the episode, the number and type of consultations, antibiotic prescriptions and complications (mastoiditis, ICPC code H74.02) were extracted. OM treated with antibiotics was defined as an OM episode with an oral or topical antibiotic prescription according to the Anatomical Therapeutical Chemical (ATC) classification. Since episodes and antibiotic prescriptions are not directly linked in the JGPN database, antibiotic prescriptions within two days before and after the start and stop date of the episode were captured. The full list of ATC codes used in this study can be found in **Supplementary Table 1**. Additionally, data on acute upper respiratory tract infections (URTI, ICPC code R74) were extracted.

### Implementation of Infection Control Measures in the Netherlands

On March 15^th^ 2020, the prime minister of the Netherlands introduced social distancing, working from home, and the closure of restaurants/bars, sport facilitates and schools/daycare. Primary schools and daycare centres reopened 11^th^ of May 2020 (Rijksoverheid, 2020), but a 1.5 meter distance rule remained in place and wearing non-medical face-masks was introduced (both not obligatory for children up to 12 year of age). The second lockdown, including closure of schools and daycare centres, started the December 14^th^, 2020 and ended 9 February 9^th^, 2021.

### Analysis

We calculated the total number of OM episodes pre-COVID-19 and during the COVID-19 pandemic. Incidence rates (IR) were calculated per 1000 person-years by dividing the number of OM episodes by the total number of person-years in that specific time period. In stratified analyses, children were split into the following age groups: <2 year, 2-6 year and ≥ 6-12 years. Differences in overall OM episodes and those treated with antibiotics between the two time periods were expressed as rate ratios (IRR) and rate differences (IRD) with accompanying 95% confidence intervals (CI). All statistical analyses were performed with SPSS (version 26.0, Chicago, IL, USA) and MedCalc for Windows, version 19.4 (MedCalc Software, Ostend, Belgium). The p-values for IRD were obtained using the Chi-square statistic, while the Exact Mid-P test was used to obtain the p-values for IRR.

## Results

### Study Population

In the pre-COVID-19 period, electronic health record data of 67,245 children aged 0-12 years were available (time point: 1 September 2019) whereas data of 67,134 children were available during the pandemic (time point: 1 September 2020). Sex and age distribution were similar across periods: 51% male, 16% aged <2 years, 33% 2-6 years and 51% ≥ 6-12 years.

### Overall OM Episodes

OM episodes declined considerably during the COVID-19 pandemic (**Table 1**).The IR per 1,000 child-years pre-COVID-19 vs COVID-19 for AOM were 73.7 vs 27.1 [IRR 0.37, 95% CI 0.35-0.39], for OME 9.6 vs 4.1 [IRR 0.43, 95% CI 0.37-0.49], for ear discharge 12.6 vs IR 5.8 [IRR 0.46, 95% CI 0.41-0.52] and for ear pain 18.1 vs 11.8 [IRR 0.65, 95% CI 0.60-0.71]. Gender (**Supplementary Figure 1**) and age-specific analyses revealed similar results, except for a less pronounced decline in OME episodes in children aged 0-2 years [IRD -0.66, 95% CI -2.50-1.18].

**Table 1:** Number of Otitis media episodes and episodes with antibiotic prescription pre-COVID-19 era and COVID-19 era, including rate ratios and rate difference.

Episodes of	Pre-COVID-19 era^a^					COVID-19 era^a^					Rate Ratios and Rate Differences			
	N *patients*	N *episodes*	IR *episodes^b^*	% *AB oral^c^*	% *AB top^d^*	N p*atients*	N *episodes*	IR *episodes* ^b^	% AB oral^c^	% AB top^d^	IRR *episodes^e^*	IRD *episodes^e^*	IRD *episodes with AB oral^f^*	IRD *episodes with AB top^g^*
AOM	67245	4959	73,7	47,4	10,7	67134	1822	27,1	46,2	14,5	0,37 (0,35;0.39)	-46,6 *(-49,0;-44,2)*	-22,4 *(-24,1;-20,8)*	-3,9 *(-4,7; -3,1)*
											*p<0,0001*	*p<0,0001*	*p<0,0001*	*p<0,0001*
Age 0 - 2	10896	1574	144,5	61,6	6,7	10243	681	66,5	62,1	4,1	0,46 *(0,42; 0,50)*	-78,0*(-86,8; -69,2)*	-47,6*(-54,6; -40,7)*	-7,0 *(-9,1; -4,8)*
* *						* *					*p<0,0001*	*p<0,0001*	*p<0,0001*	*p<0,0001*
Age 2 - 6	21731	2354	108,3	44,0	11,5	21859	743	34,0	41,0	15,5	0,31 *(0,29; 0,34)*	-74,3 *(-79,3; -69,3)*	-33,7*(-37,0; -30,4)*	-7,2*(-8,9; -5,4)*
* *						* *					*p<0,0001*	*p<0,0001*	*p<0,0001*	*p<0,0001*
Age 6 - 12	34618	1031	29,8	33,8	14,8	35032	398	11,4	28,4	30,7	0,38 *(0,34; 0,43)*	-18,4 *(-20,6; -16,3)*	-6,8 *(-8,0; -5,6)*	-0,9 *(1,9; -0,0)*
											*p<0,0001*	*p<0,0001*	*p<0,0001*	*p=0,0491*
OME	67245	648	9,6	8,5	10,2	67134	277	4,1	7,2	12,6	0,43 *(0,37; 0,49)*	-5,5 *(-6,4; -4,6)*	-0,5 *(-0,8; -0,27)*	-0,5 *(-0,75; - 0,17)*
											*p<0,0001*	*p<0,0001*	*p=0,0001*	*p=0,0021*
Age 0 - 2	10896	54	5,0	16,7	7,4	10243	44	4,3	18,2	4,5	0,87*(0,57; 1,31)*	-0,7 *(-2,5; 1,2)*	-0,04*(-0,81; 0,72)*	-0,17*(-0,63; 0,28)*
						* *					*p=0,4837*	*p=0,4819*	*p=0,9083*	*p=0,4586*
Age 2 - 6	21731	297	13,7	9,4	10,1	21859	110	5,0	7,3	7,3	0,37 *(0,29; 0,46)*	-8,6*(-10,5; -6,8)*	-0,9*(-1,5; -0,4)*	-1,0 *(-1,6; -0,5)*
						* *					*p<0,0001*	*p<0,0001*	*p=0,0008*	*p=0,0003*
Age 6 - 12	34618	297	8,6	6,1	10,8	35032	123	3,5	3,3	20,3	0,41*(0,33; 0,51)*	-5,07 *(-6,2; -3,9)*	-0,4 *(-0,7; -0,1)*	-0,2 *(-0,6; 0,2)*
											*p<0,0001*	*p<0,0001*	*p=0,0026*	*p=0,3310*
Ear discharge	67245	847	12,6	25,4	40,3	67134	388	5,8	14,2	48,2	0,46 *(0,41; 0,52)*	-6,82 *(-7,84;-5,79)*	-2,34 *(-2,9; -1,9)*	-2,3 *(-3,0: -1,6)*
						* *					*p<0,0001*	*p<0,0001*	*p<0,0001*	*p<0,0001*
Age 0 - 2	10896	222	20,4	36,9	27,0	10243	78	7,6	28,2	19,2	0,37 *(0,29; 0,49)*	-12,8*(-16,0; -9,6)*	-5,4*(-7,3; -3,5)*	-4,0 *(-5,7; -2,4)*
						* *					*p<0,0001*	*p<0,0001*	*p<0,0001*	*p<0,0001*
Age 2 - 6	21731	388	17,9	27,1	41,0	21859	158	7,2	15,8	46,8	0,40 *(0,33; 0,49)*	-10,6 *(-12,7; -8,5)*	-3,7*(-4,7; -2,7)*	-3,9*(-5,3; -2,6)*
						* *					*p<0,0001*	*p<0,0001*	*p<0,0001*	*p<0,0001*
Age 6 - 12	34618	237	6,8	11,8	51,5	35032	152	4,3	5,3	64,5	0,63 *(0,51; 0,78)*	-2,5 *(-3,6; -1,4)*	-0,6 *(-0,9; -0,2)*	-0,7 *(-1,6; 0,11)*
											*p<0,0001*	*p<0,0001*	*p=0,0008*	*P=0,088-*

a Pre COVID-19 era = March 2019 – 29 February 2020, COVID-19 era = 1 march 2020 - 28 February 2021.

b Incidence rate = total episodes per 1000 persons years.

c Percentage of episodes with an oral antibiotic prescriptions, including amoxicillin, amoxicillin/clavulanic-acid, co-trimoxazole, clarithromycin, azithromycin.

d Percentage of episodes with a topical antibiotic prescriptions, including tobramycin, ofloxacin, dexamethasone/tobramycin, dexamethasone/framycetine/gramicidin, hydrocortisone/colistin/bacitracin.

e Incidence Rate ratios (IRR) and Incidence Rate differences (IRD) per episode (Pre COVID-19 era vs COVID-19 era), including 95% confidence interval.

f IRD episodes with an oral antibiotic prescription per 1000 persons years (Pre COVID-19 era vs COVID-19 era), including 95% confidence interval.

g IRD episodes with a topical antibiotic prescription per 1000 persons years (Pre COVID-19 era vs COVID-19 era), including 95% confidence interval.

**Figure 1:**
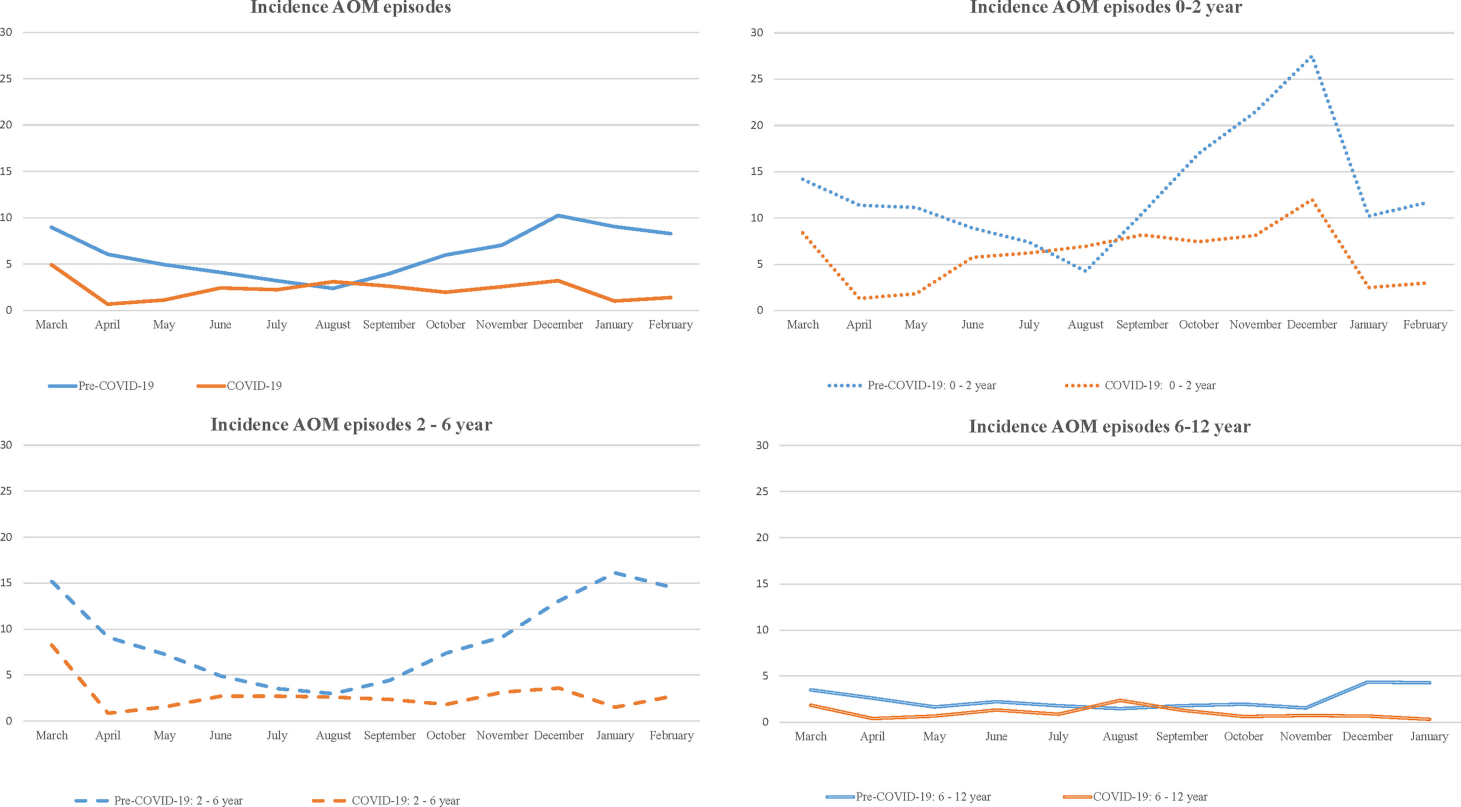
Incidence of AOM episodes per 1,000 childmonths (total and according to age) pre-COVID-19 era and COVID-19 era.

**Figure 1** shows the monthly incidences of AOM episodes per 1,000 child months for various age groups before and during the pandemic and illustrates the absence of the usual winter peak in AOM incidence during the COVID-19 pandemic, especially in young children. **Supplementary Figure 2** shows the monthly incidence of OME episodes per 1,000 child months for various age before and during the pandemic.

**Figure 2:**
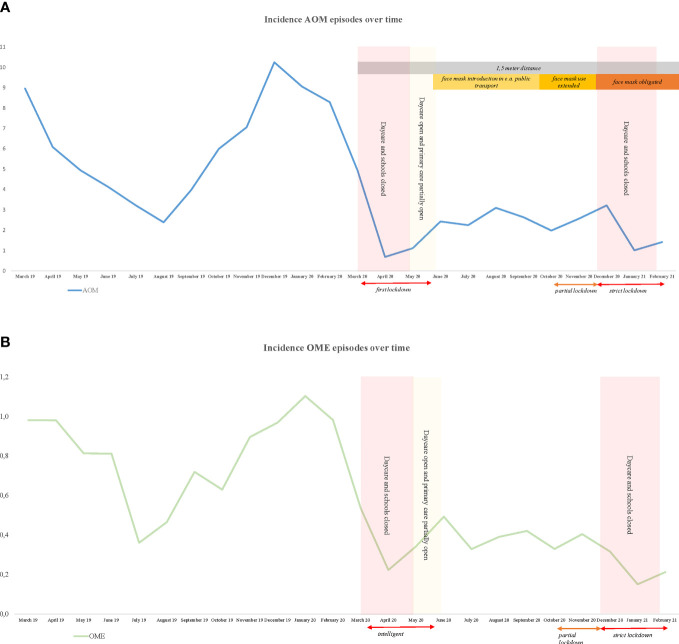
Incidence AOM and OME episodes per 1000 childmonths and government restrictions. **(A)** AOM, **(B)** OME.

**Figure 2** illustrates the timing of implementation of generic infection control measures together with the monthly incidences of AOM and OME per 1,000 child months from March 2019 to March 2021. AOM and OME incidences decrease sharply during the COVID-19 peaks as well as during closure of schools and daycare centers. Acute upper respiratory tract infections show a similar pattern (**Supplementary Figure 3**).

### OM Episodes Treated With Antibiotics

Similar to the overall OM episodes, the absolute number of AOM episodes in which oral antibiotics were prescribed declined accordingly (IRD pre-COVID-19 vs COVID-19: -22.4 per 1,000 child years), but the proportion of AOM episodes with antibiotic prescription was similar in both periods (47% vs 46%, respectively) (**Table 1**).

### Type of Consultation

The numbers of OM-episodes are based on ICPC-codes, which consist of both face-to-face and telephone GP consultations. The proportion of OM episodes (AOM, OME, ear discharge combined) which were coded based on only telephone consultation(s) only, increased over time [pre-COVID-19 vs COVID-19: 7.4% vs 22.3%].

### Complications

The incidence of acute mastoiditis remained low throughout the study period; IR per 1,000 child year pre-COVID-19 vs COVID-19: 0.15 vs 0.10 [RR 0.70, 95% 0.23-2.04].

## Discussion

This large retrospective cohort study showed that GP consultation for AOM, OME and ear discharge declined by 63%, 57% and 54% respectively in the Netherlands during the COVID-19 pandemic.

Previous studies in other countries have reported a similar trend in childhood OM incidence during the first COVID-19 peak (Hatoun et al., 2020; Aldè et al., 2021; Angoulvant et al., 2021; Iannella et al., 2021; Torretta et al., 2021). Under normal circumstances, OM typically shows a seasonal pattern with a winter peak coinciding with the increase in URTI incidence (Castagno and Lavinsky, 2002). Our study demonstrates the absence of the usual winter peak in AOM and OME during COVID-19 which is comparable with the reports of bronchiolitis from Belgium (Van Brusselen et al., 2021). The observed reduction in childhood OM might be attributed to the generic infection control measures, or changes in health care access and delivery. Although primary care services in the Netherlands remained accessible during the pandemic, the measures could have led to a higher threshold for consulting the GP, particularly early in the pandemic.

We found no evidence of an increase in the proportion of childhood OM episodes treated with antibiotics despite a substantial reduction in GP consultations for OM. This suggests that the observed decline in doctor consultations for OM was not related to OM severity and therefore not primarily attributed to a higher threshold to consultation. In line with our findings, a previous study in Scotland reported that the COVID-19 lockdown led to a decline in pediatric emergency care consultations without an associated increase in severity (Williams et al., 2021). Another recent study in the United States has also found a lower rate in respiratory infection visits around September 2020 likely attributed to the infection control measures.

The major strengths of our study are its large sample size using well-documented electronic routine primary care-based health care data. The longitudinal nature of our study allowed us to compare the same study population within the same practices during two full years, i.e. one full year pre-COVID-19 and a complete year during the COVID-19 pandemic. Some methodological limitations need to be considered. First, misclassification might have occurred. Particularly during the COVID-19 pandemic, a substantial proportion of OM diagnoses were based on telephone consultation only which could have led to misclassification in OM diagnosis. A previous study from our group found that only 50% of parent-reported OM (fever and ear pain) episodes led to a GP diagnosis of OM (Fortanier et al., 2015).However, despite the increase in telephone consultations, we found a decline in the ICPC code ‘ear pain’ as well. This suggests that our observations reflect a true decline of OM during the pandemic. Moreover, misclassification might have occurred for the ICPC code ‘ear discharge’ since we were unable to determine whether this related to an acute onset of ear discharge or chronic suppurative otitis media. Furthermore, some children presenting with AOM and ear discharge will likely be classified by their GP as ‘AOM’ [ICPC code H71], i.e. those with AOM who were prescribed topical antibiotics. Second, we were unable to reliably extract data on specialist referrals and data about out of hours primary care were not available. Therefore, our results regarding complications should be interpreted with caution. Reassuringly, a previous study from Italy did not find a significant difference in OM-related complications during the first COVID-19 wave (Torretta et al., 2021) Finally, we were not able to link reductions in childhood AOM episodes to changes in causative viruses and bacteria over time. Such data would have allowed us to better explain our observations.

### Implications

The observed decline in OM incidence similar to those observed in other common respiratory infections during the COVID-19 pandemic (Hatoun et al., 2020; Angoulvant et al., 2021; Kaur et al., 2021; Barschkett et al., 2021) providing further evidence for social interactions an hygiene as risk factors for OM. Continuing infection control measures like frequent handwashing (Little et al., 2015) may have a lasting effect on OM incidence beyond COVID-19 pandemic.

Although we should keep in mind the importance of social contacts in the development of children when considering implementing these infection control measures.

## Conclusion

GP consultation for AOM, OME and ear discharge declined by 63%, 57% and 54% respectively in the Netherlands during the COVID-19 pandemic. Similar antibiotic prescription rates before and during the pandemic indicate that the case-mix presenting to primary care did not change considerably. Our data therefore suggests a true decline as a consequence of infection control measures introduced during the pandemic.

## Data Availability Statement

The data analyzed in this study is subject to the following licenses/restrictions: Data are not publicly available due to ethical and legal restrictions. Requests to access these datasets should be directed to not applicable.

## Ethics Statement

Ethical review and approval was not required for the study on human participants in accordance with the local legislation and institutional requirements. Written informed consent from the participants’ legal guardian/next of kin was not required to participate in this study in accordance with the national legislation and the institutional requirements.

## Author Contributions

Conceptualization AS, RV, and SH. Methodology, RV, SH, AP, and AV. Formal analysis, SH. Resources, RD. Writing— original draft preparation, all authors. Writing—review and editing, all authors. Visualization, SH. All authors have read and agreed to the published version of the manuscript.

## Funding

This work is supported by a research grant from the Netherlands Organisation for Health Research and Development (ZonMw) [Rational Pharmacotherapy 5th Open Call grant number 84801 5006]. The funder has no role in design, conduct and report this study.

## Conflict of Interest

The authors declare that the research was conducted in the absence of any commercial or financial relationships that could be construed as a potential conflict of interest.

## Publisher’s Note

All claims expressed in this article are solely those of the authors and do not necessarily represent those of their affiliated organizations, or those of the publisher, the editors and the reviewers. Any product that may be evaluated in this article, or claim that may be made by its manufacturer, is not guaranteed or endorsed by the publisher.
