# Supplementary material for: A Strong Decline in the Incidence of Childhood Otitis Media During the COVID-19 Pandemic in the Netherlands
Source: Front Cell Infect Microbiol. 2021 Nov 1;11:768377. doi: 10.3389/fcimb.2021.768377 (PMC8591181; doi:10.3389/fcimb.2021.768377)
Supplement: Supplementary file 1 [file DataSheet_1.docx]

Supplementary Material

**Supplementary Table 1**

Oral and topical antibiotics with Anatomical Therapeutic Chemical (ATC) codes

| Oral antibiotics | ATC codes |
| --- | --- |
| *amoxicillin* | *J01CA04* |
| *amoxicillin/clavulanic-acid* | *J01CR02* |
| *co-trimoxazole* | *J01EE01* |
| *clarithromycin* | *J01FA09* |
| *azithromycin* | *J01FA10* |
| Topical antibiotics | ATC codes |
| *tobramycin* | *J01GB01* |
| *ofloxacin* | *S01AE01/S02AA16/S01AX11* |
| *dexamethasone/tobramycin* | *S01CA01* |
| *dexamethasone/framycetine/gramicidin* | *S02CA06* |
| *hydrocortisone/colistin/bacitracin* | *S02CA03* |

**Supplementary Figure 1. Incidence of OM episodes per 1000 childyears (according to gender and age) Pre-COVID-19 era and COVID-19 era.**

**Supplementary Figure 2. Incidence of OME episodes per 1,000 childmonths (total and according to age) Pre-COVID-19 era and COVID-19 era.**

**Supplementary Figure 3. Incidence AOM, OME and URTI episodes per 1000 childmonths and government restrictions**

Daycare and schools closed

Daycare open and primary care partially open

AOM; acute otitis media, OME; otitis media with effusion; URTI; upper respiratory tract infection
